# Addressing Risk Perceptions of Low-Dose Radiation Exposure

**DOI:** 10.1177/15593258221088428

**Published:** 2022-04-06

**Authors:** Margot Hurlbert, Larissa Shasko, MIchaela Neetz

**Affiliations:** 1 University of Regina, Regina, Canada; 2 Johnson Shoyama Graduate School of Public Policy, Saskatoon, Canada

**Keywords:** radiation, low-dose radiation, linear no-threshold, risk perception

## Abstract

Concern over low-dose radiation (LDR) (exposure of less than 100 milligray (mGy)) is resulting in people refusing diagnostic procedures and medical treatment^1^ and also inhibiting revision of the linear no-threshold (LNT) assumption that informs much of science policy. This article reviews representative surveys in Ontario and Saskatchewan and focus groups conducted with science and policy stakeholders in addressing how the public and policy stakeholders understand issues of exposure to LDR and how policy issues can be addressed.

Research results from focus groups demonstrated that policy stakeholders are knowledgeable about issues surrounding the public and perceptions about LDR and implications for policy consistent with LDR literature. Participants understood that the challenge went beyond providing more education about LDR and issues of emotions and biases must be addressed. This research resulted in rich suggestions for public communication and engagement surrounding LDR and a process for addressing the issue of the LNT.

## Introduction

It has long been known that the public has concern in relation to radiation exposure, that is not shared with nuclear experts.^1^ These concerns include that all radiation exposures (including X-rays) are harmful (i.e., carcinogenic), radiation exposures are cumulative within our body, and children are more susceptible to radiation.^2^ A common thread between problems of radiation worker exposure during cancer treatment and keeping health care workers in these areas safe is the issue of exposure to low-dose radiation (LDR) (defined as below 100 mSv (the radiation protection policy definition. However, a radiobiology definition of LDR is from the background radiation level to the dose rate threshold for the onset of lasting detrimental health effects).^1^ Hendee (1991)^3^ concluded that the news media and entertainment industry have contributed to the public’s concern and abhorrence of radiation, radioactivity, and nuclear energy. Public perceptions have long been acknowledged as being complex and influenced by many factors such as value systems, politics, media (the news and entertainment industry motivated by “health scare stories about” exposures to radiation, and clear communication of scientific information. This concern has had policy implications, including refusal to accept LDR health interventions such as X-rays,^4^ even by doctors^5^ with real effects of trauma caused by fear of future cancer.

This article answers the question of how the public and policy stakeholders understand issues of exposure to LDR and how LDR perception issues and the LNT presumption informing regulation^2^ should be addressed. Research results include a representative survey in 2 Canadian provinces and focus groups with policy stakeholders at a collaborative interactive session at the Canadian Science and Policy Conference tackling these issues.

### LDR Exposure, Policy, and Public Perceptions

Mostly, people are exposed to LDR that is naturally occurring. However, people voluntarily expose themselves to LDR when taking air flights and during health interventions including medical and dental X-rays, testing for medical conditions such as Parkinson’s, and cancer treatments. In the United States, the National Council on Radiation Protection and Measurements (NCRP)^6^ concluded in 2009 that naturally occurring radon accounted for 37% of LDR exposure, naturally occurring radiation (other than radon) 13%, medical imaging 48%, and airplane travel 2% (with nuclear power plants constituting 0%).

Radiation is a weak carcinogen at high doses. However, there is no evidence that radiation is a carcinogen at any dose below the threshold dose for the onset of lasting detrimental health effects or that radiation exposures to background levels are damaging to health.^7-9^ However, a linear “no-threshold” (LNT) assumption (conceptualized in the 1950s) currently exists in relation to exposure to radiation either at work (when employed in occupations in the medical field where exposure occurs on a daily basis), or in relation to proximity to nuclear plants (when establishing disaster and emergency planning areas in proximity to nuclear installations). The linear “no-threshold” assumption posits that exposure to radiation can only be detrimental and the health risks resulting are linearly proportional to exposure dose.^10^ This policy was developed based on biological mechanisms through which radiation exposure can induce harm and ignores biological evidence that every organism has powerful adaptive protection systems that prevent damage, repair damage, remove damage, and restore health.

Cuttler and Calabrese document how the Rockefeller Foundation, which had been funding and managing the U.S. National Academy of Sciences (NAS), initiated a study in 1954 on the genetic effects of radiation.^1^ The study, published in June 1956, recommended that the LNT dose-response model be used to assess the risk of radiation-induced genetic mutations instead of the threshold model, which had been the basis for the “tolerance dose” rate limit the radiologists had employed for their protection, for more than 3 decades.^1^ This LNT recommendation was controversial because it was based upon flawed research on fruit flies. That research was contradicted by the 10-year study of about 75 000 children of the atomic bomb survivors that showed no evidence of hereditary damage. The NAS, however, disregarded this crucially important human evidence.^1^

Calabrese^11^ further documents the NAS study was immediately followed by a deeply flawed study of the incidence of leukemia among the atomic bomb survivors. Published in 1957, it suggested a link between any exposure to radiation and a risk of cancer by fitting the LNT model to the data. A revisit of this study in 2015 revealed that the author had combined the data in the low-dose zone with the data in the control zone, which concealed the evidence of the high threshold, at 1.1 Gy, for the onset of radiation-induced leukemia. The 32 700 survivors in the low-dose zone, whose exposures were below this threshold, had a lower-than-normal incidence of leukemia.^1^ In 1959, the NCRP adopted the precautionary principle policy, which, in effect, meant that the LNT model would be employed to estimate the risk of radiation-induced cancer. This decision, published in 1960, was based on public fear and lack of knowledge. The United States and essentially all other countries followed this lead.^1,11^

The LNT risk and regulatory burden has been questioned since the 1980s and recently by the World Nuclear Association who called for the adoption of an all-hazards approach, placing different risks in perspective and the appropriate context in line with the latest scientific evidence.^12^ An alternative theory to the LNT is the “hormesis” theory that posits that the existence of a threshold dose for the onset of harm and that doses below the threshold dose have no latent risk and may have health benefits. Several scholars have refused to endorse the LNT model.^13-15^ Several other academics go so far as to state that the LNT model cannot be scientifically valid.^16,17^ Calabrese^18-20^ documents the historic development of the LNT as a policy and questions whether there ever was a scientific basis for the policy. Evidence suggests that at low doses, there is an absence of biological detriment and may even be a beneficial effect following exposure to LDR. It is now widely accepted that radiation also produces a wide range of epigenetic effects, effects on inflammatory processes, and effects on the cellular immune system.^5^

The uncertainty of the regulatory landscape, and evidence to suggest that the current low-dose toxicity paradigm is in error, heightens the need for more social science research, but also more engagement in the science, public, and policy community. Social science continually reinvents versions of the public deficit explanation (that scientists simply need to speak truth in science to fill the information gap) to address this misunderstanding and correct.^21^ Wynne notes that the problem is due to a continuing failure of scientific and policy institutions to place their science and institutional culture into a dialogue that is open to question, debate, and revision. Douglas,^22^ however, points to the chronic uncertainty in scientific inquiry and hypothesis testing that might lead to revision, especially in relation to predicting the future. While science is striving for timeless truths, policy and politics works in the mess of conflict, change, and limits, and these differences ultimately end in a profound entanglement, but this entanglement is not without resolution.

Social science literature has considered these issues. Understanding how people think about and respond to risk is fundamental to policymaking in relation to health, safety, and hazards.^23^ Fear or dread, uncertainty, and time frame (immediate vs long term) of consequences of radiation exposure impact people’s perception of risk; who communicates information relevant to risk construction and the source of information are key determinants of people’s perceptions of the veracity of information.^24^ As the degree of benefit associated with the exposure increases, the degree of risk acceptance increases, thus explaining why exposure through medical interventions is generally accepted. In an effort to deepen the understanding of perceived risks, research has turned to explore the concepts of credibility and trust, asking who is trusted to communicate.^25^ The concepts of trust and credibility are not independent of each other and are often defined differently by different authors. The public filters information making a judgment partially dependent on the perceived credibility of the information source. Credibility is defined as “the quality or power of inspiring belief.”^26^

Media has been shown to amplify negative imagery and influence trust.^27,28^ As trust has been observed to be asymmetric in the case of nuclear energy (meaning it is easy to lose but hard to regain),^1^ the influence the media has on trust is crucial. Greenberg^29,30^ concludes a genuine concern can be captured, amplified and enhanced by television and print media. The idea “no news is good news” amplifies negative media coverage and may further embed existing beliefs or leave those who are undecided or somewhere in between the poles in their views more confused or skeptical.

Ideally, the uncertainty connected to risk in decision-making is best navigated through interactive methods of engagement where both analytical and experiential systems of thinking can be utilized.^31^ While experts think of risk in relation to “hazard,” the public think of risk associated with nuclear issues in terms of “dread” and “outrage.”^32^ Methods to engage with analytical and experiential systems of thinking that move past emotional initial responses to difficult risk issues include participatory decision-making that is facilitated in group settings.^31^ Furthermore,^33^ deliberation tools can be used to nudge decision-making away from heuristics and judgment biases.^33^ Deliberation tools are based on prompting individuals to reflect more deeply and take an active role in decision-making. In group settings, deliberative dialogues can be used to encourage exploration of differences of opinion without debate or silence taking over. Facilitating group discussions in a deliberative dialogue setting can help those who hold different opinions better understand each other and improve communication among stakeholders when knowledge gaps exist.

## Method

This article is based on a concurrent mixed-method study that employed focus groups and a representative telephone survey in Saskatchewan and Ontario. The telephone survey was conducted between November 2019 and July 2020.1104 respondents participated in Saskatchewan and 1008 in Ontario, representing the geographical dispersion of the provincial populations, gender, and Indigenous status. While older respondents were the most numerous (65–75) followed by (55–64); younger respondents were less represented. 72 focus group participants participated in Ottawa, Canada, in November 2019.

Focus group discussions were organized at the 2019 Canadian Science Policy Conference (CSPC) to explore the question of how policy stakeholders understand this issue of different perceptions of exposure to LDR and how it should be addressed. The annual conference took place in Ottawa, Ontario, on November 13, 14, and 15, 2019 which was prior to completion of the survey. Focus groups are a qualitative method that facilitate the study of explanatory research questions that ask “why” and “how.”^34^ Focus group discussions provide qualitative data within a social context where ideas can be expanded upon by the group.

The CSPC attracts a multisectoral and interdisciplinary audience to present and discuss current issues of science and innovation policy. The CSPC’s audience is policy bureaucrats in government as well as policy professionals in industry, academia, government, and non-profit organizations. Executives and senior management account for nearly half of the delegates in attendance.^35^ Bureaucrats and policy professionals have considerable formal policymaking authority and play an important, and often overlooked, role in the policy process.^36^ Policy analysts in the bureaucracy regularly perform duties such as identifying policy issues, identifying and assessing policy options, and conducting policy-related research, networking with policy stakeholders, and data collection.^37^ Because of this, this contingent of policy actors has a large influence on the policy agenda of government.^38^

The November 13 CSPC panel session titled “Risk, Uncertainty, Unknowns and Nonsense – Engagement with the Public on Radiation, Nuclear, and Climate” attracted 72 participants. This session explored public perceptions of LDR in relation to new research that challenges the current linear toxicity paradigm. The panel session included a brief presentation surrounding LDR, 7 break-out focus group discussions, and a final full group debrief. The presentation reviewed facts surrounding types of radiation, ionizing and non-ionizing radiation, background radiation Canadians are exposed to, and the LNT policy and evidence negating it.

The focus group discussions were also prefaced with guidelines for deliberative dialogues. Radiation is often associated with subjects such as nuclear power that can polarize debates. The discussions were framed to participants as deliberative dialogues in an effort to mediate this effect and to encourage participation by creating a safe space. The 7 groups were mediated by one of the research team members and guided by the 4 topics (Table 1) identified as relevant based on the literature and also the general outline agreed to in the research team’s ethics application. However, discussions were open-ended allowing participants to discuss issues most relevant to them. Focus group discussions were recorded, transcribed, and then coded using the categories of questions asked in Table 1, but also searching for emergent themes. Because of the CSPC context, participants in all focus groups discussed both personal views and public views and policy implications.

**Table 1.** Guiding Questions for Focus Groups.

Discussion topics	Questions
A. Perceptions of LDR	1. What is your perception of LDR? 2. On balance, do the benefits of LDR outweigh its inherent risks to human life? Why?
B. Trust in LDR information sources	3. Which source of LDR information is trusted the most? Why?
C. The role of the media in influencing perceptions of LDR	4. Which media (e.g., TV, radio, newspaper, and internet) is the most trustworthy source of information about LDR? Why? 5. How important are social media networks such as Facebook and Twitter, as a source of information about radiation? Why?
D. Recommendations	6. How can and should the LNT assumption in relation to LDR be modified?

As attendance to this panel was voluntary, the control of focus group size was limited and ranged from approximately 9–14 participants per group. When appropriate, facilitators asked questions or provided information to clarify ideas and gather more in-depth information. After the discussion, the panel debriefed as 1 larger group discussing the overlaps and divergence of the focus group conversations. Focus groups were audiotaped and transcribed for analysis and then coded and analyzed to determine themes and understandings.

## Results

While there was no consensus amongst the 72 participants in relation to these questions, there were some interesting emergent themes and novel ideas. The overarching research question was how the public and policy stakeholders understand issues of exposure to LDR and how this issue can be addressed. Policy stakeholders in the focus groups also discussed the LNT, its implications and issues for policy, and possible resolution. Analysis of the focus group discussions revealed the emergence of 4 key themes. The first acknowledged LDR as a reality, a part of everyday life and the natural world. Second, participants felt strongly that the public has misperceptions and emotional responses to the issues of LDR. Third, trusted sources of low-dose information have vested interests, and to resolve this issue, the practice of transparency and public dissemination is crucial. Fourth, LDR generally lacks a positive voice, and scientists could help fill this gap.

### Perceptions of LDR

There were 3 main findings in relation to LDR and public perceptions arising from the survey and the focus groups. These findings concerned confusion surrounding LDR, decision-making, and the benefits and risks of LDR. First, the public and policy stakeholders have misunderstanding, confusion, and concern surrounding LDR. This conclusion is supported by several sources of data. First, published and accessible research rarely discusses LDR. A Google search of the PubMed database determined that only 3.7% (33 952 of a total of 922 113) of articles on radiation made mention of LDR.

**Figure 1.**
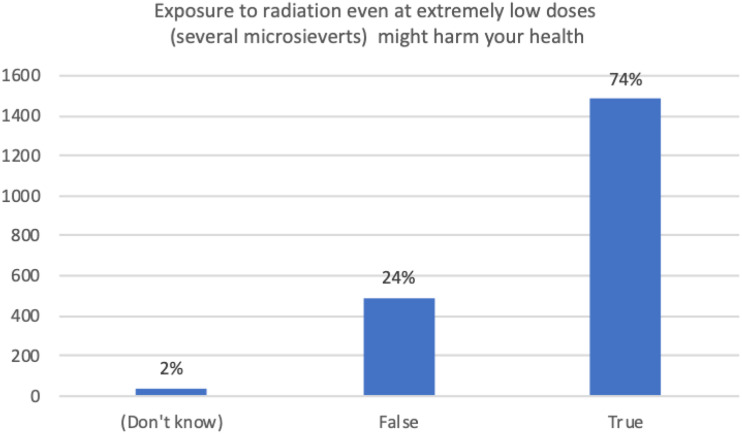
Exposure to radiation even at extremely low doses (several microsieverts) might harm your health?

Second, our survey results confirmed that most of the general public are not familiar with what LDR is and where they may be exposed to LDR. Figure 1 shows that a majority of people surveyed (over 70%) believed that exposure at extremely low doses (several microsieverts, which is well below the threshold for LDR of 100 mSvt) might harm health.

Third, within the CSPC policy stakeholder focus groups, there was also confusion and discussion surrounding ionizing vs non-ionizing radiation exposure and lack of knowledge of what a microsievert was, even after a presentation that provided this information. Focus group participants confirmed that addressing this confusion will be no easy task. One participant relayed that he had taken physics and a specific university class in nuclear physics and did not know the answer to the question whether exposure to less than 100 microsieverts was safe or not.

While focus group discussions confirmed confusion surrounding specifics of what low doses are and their precise definition, many focus group participants recognized that public perception of risk is complex, in alignment with the literature. Focus group participants described public perceptions of the risk of radiation as a “funny” area. Analogies were drawn to bananas since bananas contain naturally occurring radioactive isotopes (potassium-40), and eating 1 banana is approximately 1% of the average daily exposure to radiation of 100 banana equivalent doses (BED). One participant stated that the best way to educate about LDR is to discuss bananas because discussing units of measurements and X-rays is far too technical and issue laden. That person stated, “It’s inconsistent to tell people that X-rays are harmless but advise them they have to wear a large heavy lead blanket for protection.”

Some examples raised in discussion included how many people voluntarily choose to expose themselves to radiation, including sunburns (which are harmful but many do not bother to use sunscreen) and cigarettes (a huge radiation source), yet people smoke. These examples lead to a great deal of discussion surrounding decision-making.

Decision-making is not simply a rational process where analytical, carefully considered thinking occurs. Heuristics and judgment biases influence how decisions are made, with heuristics serving as mental shortcuts and judgment biases operating without awareness of their influence.^1^ Heuristics ground opinions and form path-dependent perceptions that are difficult to move once established. Heuristics connected to radiation are often anchored in seeing radiation as a product of nuclear energy rather than nuclear energy as a product of radiation. 25% of survey respondents felt radiation from nuclear power facilities was dangerous or very dangerous while 45% slightly and moderately dangerous.

**Figure 2.**
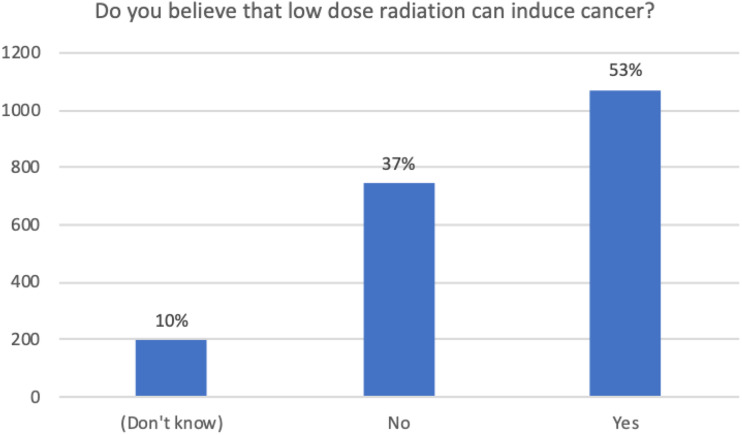
Do you believe that low-dose radiation can induce cancer?

Medical applications are often anchored in the opposite understanding due to the benefit accruing to the individual undertaking them.^39^ Medical applications such as diagnostics and cancer treatments are perhaps more easily understood as a use of radiation. Figure 2 shows that a majority of people believe that LDR can influence cancer. Interestingly, the vast majority of respondents are prepared to be exposed to this risk in relation to medical treatment. Just over 50% of respondents surveyed believed chest x-rays were slightly or moderately dangerous, 9% believed they were dangerous and very dangerous, and 36% believed there was no or little danger (Qs 43–50). However, most people voluntarily accessed medical diagnostics; only 15% surveyed had ever declined to undergo an x-ray examination. Many focus group participants expressed similar sentiments to these findings of the survey.

Hendee (1991)^3^ concluded this is in part due to people’s emotional response to involuntary risk exposure (as opposed to a voluntary assumed risk whereby an individual assumes responsibility for a beneficial or adverse outcome). Individuals are making decisions in terms of air travel and medical diagnostics on a daily basis. Travelers voluntarily assume exposure in their travel and via airport scanners. In moments where an immediate decision needs to be made to accept or reject exposure (ex. Airport scanner), there may not be time to weigh the benefits and risks leading to a decision to reject it, especially if the benefit is for the public collectively and not directly benefiting the individual decision maker (ex. Airport scanner benefits air traffic security for the public vs an X-ray benefiting you and your broken bone). This finding affirms the theory that as the degree of benefit associated with exposure increases, the degree of risk acceptance increases, thus explaining why exposure through voluntary exposure such as medical interventions is accepted.^39^

Most of the focus groups were in agreement that basic education would be worthwhile. The best communicators to assist with individual decision-making were identified as knowledgeable health experts without conflict of interest, such as a community physician. However, the communicator must first respond to perceptions of risks and underlying emotions, as facts often fail to counteract fears. Informing people of the very low level of risks from diagnostic radiation was proposed in order to curtail apprehension and advance better dialogue between doctors and patients. Patient-doctor decision-making was felt by participants as most importantly based on best evidence, the unique needs of the patient, and the expertise of the physician, not radiophobia.^2^

**Figure 3.**
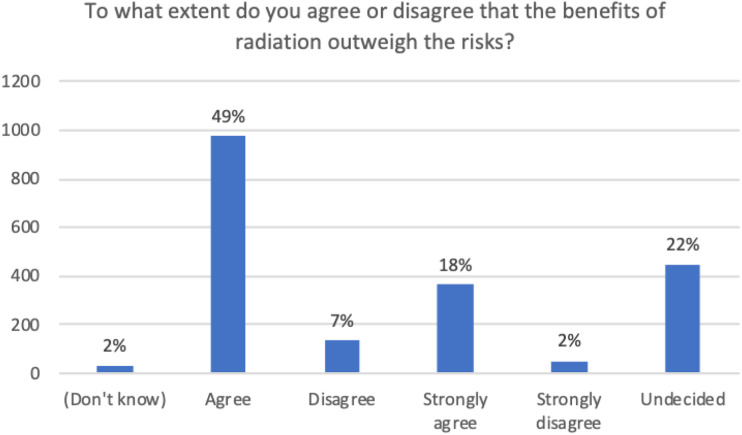
To what extent do you agree or disagree that the benefits of radiation outweigh the risks?

**Figure 4.**
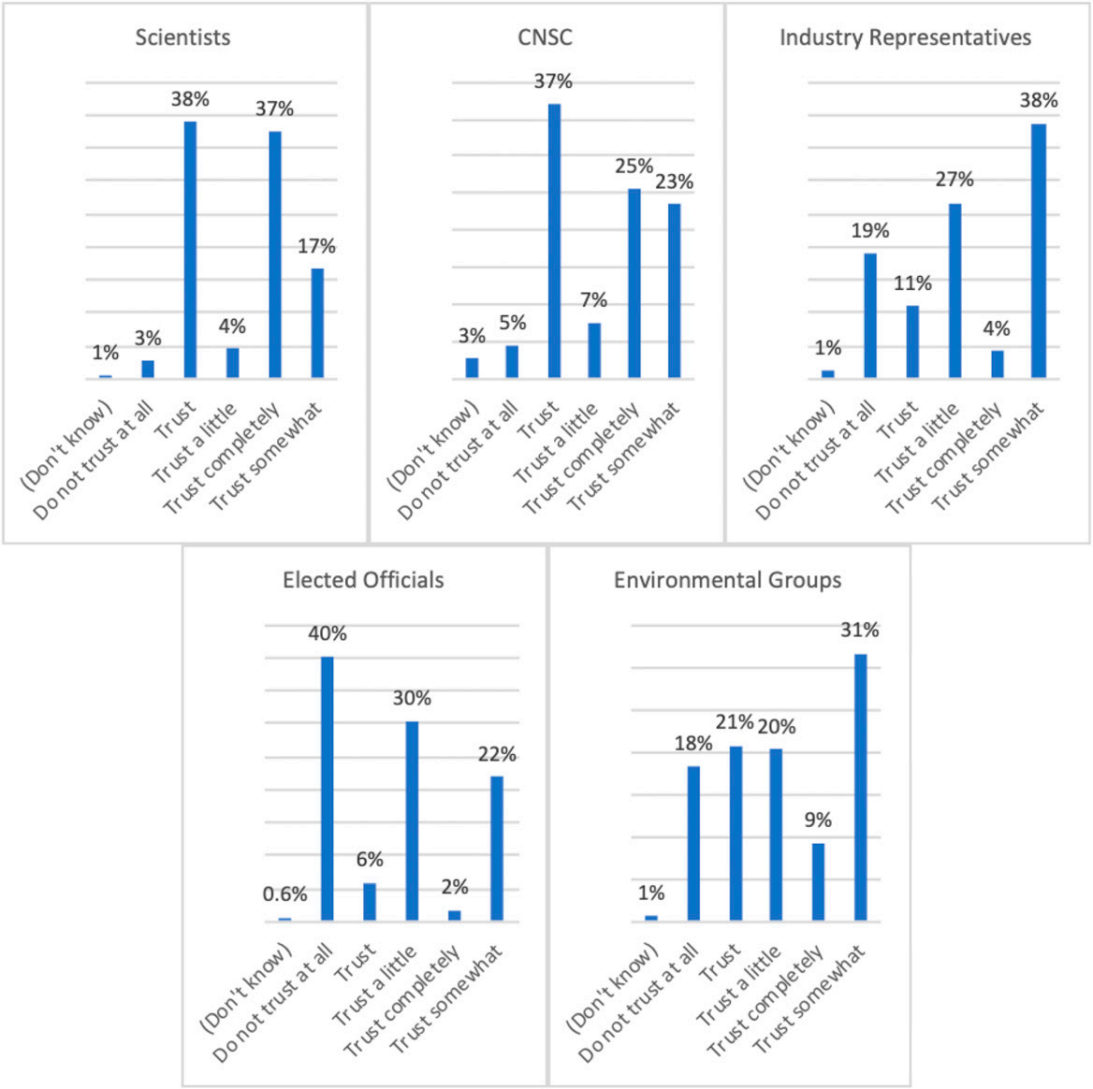
Who do you trust as a credible source of information when you hear about radiation?

**Figure 5.**
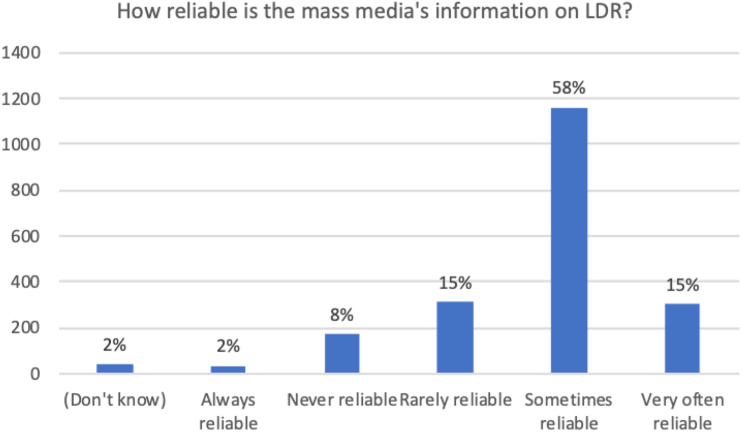
In your opinion, how reliable is the mass media’s information on low-dose radiation?

When asked about the balance of benefits and risks, groups diverged into 2 different paths of reasoning. Several groups discussed that the distribution of benefits and risks is context-dependent. The example used to demonstrate this distribution was living in proximity to a nuclear power plant, which was regarded as having a benefit of producing clean energy vs a nuclear waste facility, which was regarded as not having the same benefit of a nuclear power plant. This view opened a discussion of chosen vs imposed risks. Several other focus groups concluded that the balance of benefits and risks is a non-issue. These participants perceived LDR as inescapable in how society has chosen to construct life. Research results were surprising as there was no discussion of “inherent” risks and a consensus was achieved that the benefits outweighed any risks (Figures 3-5).

### Sources of LDR Information

Both the survey and the focus groups (discussion topic B) investigated who was trusted to communicate about LDR and how. Survey respondents ranked trust in scientists highest as a credible source for information about radiation, followed by the Canadian nuclear regulator, with only 6.5% not trusting scientists and 11% not trusting the regulator. Trust was mixed in relation to environmental groups and industry representatives, with the latter skewing more into the “not trusting” side. 65% did not trust elected officials as a credible source of information about radiation. These findings are consistent with the findings of Greenberg (2013)^29^ in the United States, where in general, independent scientists and regulators were considered to be the most trusted, while the media and U.S. Congress the least.

In the focus group discussions, participants stated that it was clear that if the person who was the source of information had an interest in the matter, they would not be perceived as trustworthy. The industry was perceived as biased but would be considered credible if there was an external review or oversight. Those sources which were mentioned as being credible represent only a small window of trust. These included scientists and medical professionals. Ho et al. (2018)^40^ used source credibility theory to investigate what sources of nuclear energy information and which stakeholders were perceived as credible by the public. Similar to other authors, they conceptualized 2 key dimensions of credibility: source expertise and source trustworthiness. Source expertise refers to the authority, knowledge, and capabilities toward a particular subject matter that a source is perceived to have.^41^ Source trustworthiness refers to the degree of acceptance of a source’s honesty, integrity, and message content accuracy.^42^ This was confirmed in the focus groups. It was noted that there is a value gap, meaning the information will be rejected if it does not come from a source that already meets the individual’s values. For instance, a scientist for an environmental group would have less credibility than a university tenured scientist.

One focus group participant, an Indigenous cancer researcher, stated that in his Indigenous community, scientists might not even be regarded as trustworthy. He identified the situation in Grassy Narrows and how the community has not had safe drinking water for the past 35 years and as a result are highly suspicious of scientists and government communication. His suggestion was that scientific communication had to be very specific for each community and culturally, geographically relevant.

The medium through which information is presented also influences how trustworthy it is perceived. Focus group participants stated that the only source of truly trustworthy information was scientific journals. In contrast, survey respondents identified their main source of information about LDR (from most accessed to least) as the Internet, television, education institutions, newspapers, CBC radio, friends and family, and magazines.

60% of survey respondents considered the mass media as sometimes reliable respecting information on LDR (with 10% stating mass media was “never” reliable). Focus groups also discussed the role of social media in information sharing (a question that was not asked in the survey). There was a fairly negative reaction overall to finding information on social media. Interestingly, certain niches of social media were identified where it can be used as a tool for sharing information and dispelling misinformation. Focus group participants identified that if social media use centers around science sharing groups, it is more likely to see reliable information sources online. Additionally, there are Facebook groups that require academic citations with the posting of information, which filters out some of the inaccuracies. Prabhu and Rosenkrantz (2015)^43^ recommended more active engagement on Twitter by radiologists and physicists to disseminate more balanced information on CT scan radiation risks and dispel inaccuracies they had discovered in their review of the quality of information and perspectives in Twitter posts on the subject.

Focus group discussions also ventured into how LDR information should be presented. There was broad support for the idea that trust increases when information is presented openly and in public mediums, allowing individuals to make their own decisions and when 1 party does not try to convince or persuade. Transparency is important for credibility. One participant shared how transparency is key in respect of nuclear information in France where nuclear energy is predominant and accepted. If framed as a debate, it is important to have information sources that acknowledge and explain why they take a differing position, or a scientist that can explain the differences. Information and data should also be presented in a balanced way acknowledging any shortcomings.

Focus group participants pointed out that admitting uncertainty is important. However, providing current and accurate information is equally important to not add to any confusion around the topic. An example was shared in one of the focus groups where contradictory information on acceptable levels of radon was presented by a local newspaper, Health Canada, a lab (SNOLAB), in mining, and by an international radiation center. There was a resolution of this issue in the group that is expanded on in 4.3.

### Policy Recommendations Surrounding LDR

In addition to the policy recommendations stemming from the findings in 4.2, the focus group participants developed some novel suggestions. Focus group participants did not achieve consensus surrounding the resolution of the LNT presumption and countering hormesis theory. Only 1 participant in the focus group identified themselves as knowledgeable about hormesis as a result of LDR; participants did ask why hormesis was not more widespread knowledge. One focus group participant pointed out that it would be useful to resolve the LNT presumption and used the example of malachite green. Currently, any part per trillion is considered unsafe. Because of better detection equipment, when this substance is detected in fish, the fish are sold outside Canada. The participant noted that selling fish that are unacceptable in Canada to another country is not equitable. Similar repercussions for changing the LNT only in Canada were identified by this focus group as potentially problematic.

There was strong support that the Canadian nuclear regulator and the scientific community, together with the international scientific community should resolve the debate, prepare guidelines that could then be adopted and disseminated in the science and policy space. There was no expressed disagreement for this being an internationally explored and nationally regulated issue. However, participants did not endorse the view that the public did not care and experts should just unilaterally make the decision; as identified by Lave et al. (1989), a better strategy was to address public perceptions with honest and accurate scientific information.

After discussion of the LNT issue, a communication strategy in relation to LDR information was developed. One of the strongest recommendations was that there were very many missed opportunities where those with experience and education could be sharing their knowledge. Mainstream media was identified as portraying radiation with a negative outlook. TV series like Chernobyl and the Simpsons should be seen as opportunities to open discussions. Just as climate scientists have taken the initiative to counter any climate change denial and ensure the public is not misinformed through their own appearances in the media and international bodies such as the Intergovernmental Panel on Climate Change, so should scientists and health care workers who work with LDR. Novel methods of communicating about LDR that move beyond 1 industry and sector were believed to be required. A mining focus group participant recounted how they had developed information surrounding LDR, but when they tested the reception of the public to the LDR it was so negative that they have never moved forward with their education project.

In 2 focus groups discussing the Chernobyl series, 1 participant claimed it was accurate and other participant(s) who had disclosed their education and were seen as credible in the group pointed out some of the inaccuracies in the series. This led to the following question: “If nuclear scientists know the movie, and possibly other movies, aren’t accurate, why don’t they inform the public?” This points to the lack of credible voices participating in this space. Additionally, positive messaging is lacking when there are no any “burning” LDR issues in the media. One participant stated, “positive media doesn’t really exist as the positive news is that nothing is happening, the nuclear power plants are working away without any incidents.” In another focus group, 1 participant described public outcry when weapons-grade plutonium was to be transported by helicopter to a reactor site for storage. What the participant identified as a good Samaritan safety storage service was stymied when the actual radioactive exposure of the material “could be carried around for half a year in your pocket and you would not receive even half the allowable dose of radiation.” In respect of both these incidents, focus group participants felt that the nuclear industry was either afraid to engage with the public, apathetic about engaging with the public, or dismissive that the public was competent to engage in LDR science.

Several focus groups discussed stories where significant misinformation surrounding events was reported in the media, or communicated to the public worsening public fears surrounding LDR. One story was shared by participants in more than 1 focus group. This story related to the distribution of potassium iodide pills to a larger radius of residents living near nuclear power plants in Ontario. It is recommended that stable potassium iodide be stocked by individual homeowners in the unlikely case of a potential nuclear incident. However, at the time of distribution of the potassium iodide, little information was given to the general public, leaving many fearful. One participant said the lack of clarity and transparency from the nuclear facility and health officials caused considerable distress to the participant and their neighbors. Another participant (a nuclear power plant worker) spoke about preconceived notions they had heard expressed about this. They confirmed the distress caused in the community and with their neighbors because of this dissemination. They said they could tell their neighbors 1,000 times that this is just an extra regulation, 1 more safety precaution in the many precautions already taken, and that everything is fine; there is no need to worry. But still, the neighbors will not believe them and will be unsure because of the manner the stable potassium iodide was distributed.

Participants in these discussions concluded that information surrounding LDR needs to be shared proactively, and not in a reactive defensive manner. There was broad agreement that the best audience is youth, and not adults. One participant stated, “If people have already decided that something is bad, anything you say is regarded as spurious.” This experience is consistent with Kim (2014)^39^ and the finding that negative impressions that are subjectively formed cannot be countered by the presentation of a couple arguments negating them. However, radiation is used in many fields including health screening, food processing, and medical treatment,^44^ and messaging surrounding these uses can help establish a better balanced view of radiation.^39^ Focus group participants also recommended that framing the benefits of LDR is very important as there was too much messaging surrounding risk, danger, and protection surrounding LDR. As concluded by Hendee (1991),^3^ communicators should be expert and independent, uncertainties and estimates in data should be transparently disclosed, health risks should be balanced with information about the benefits of taking the risk or the health risks associated with not accepting the technology, and risks should be compared with those when the technology was not available.

Focus group participants agreed that 5 different positive nuclear information dissemination were required to counter 1 negative media dissemination; participants believed Canada should promote its nuclear radiation medical program and relate it as a by-product of the nuclear energy industry. One focus group participant pointed out that they worked with nuclear waste management, with and near nuclear substances all the time, and were not worried about health and safety. Other participants advised that even a simple statement like that from someone in the industry could greatly influence public perception.

## Conclusions

Based on survey results and augmented by focus groups conducted with science policy stakeholders, this research determined that communication is an important response to confusion and misunderstanding concerning LDR. Policy stakeholders have a unique perspective on policy options and influence the policy agenda. As policy stakeholders participating in focus groups had a grasp of LDR and LNT issues consistent with the literature, their thoughts on addressing public perceptions of LDR offer unique insight.

Policy stakeholders were adamant that simply having an expert fill the deficit is not the solution. Focus group participants supported the idea that people with expertise in LDR, either scientists or people working in the area, have a positive obligation to provide information and engage with the public in order to counter misinformation in the public realm about LDR and are the most trusted source of information. (Calls for an increasing role for independent scientists fulfilling this function are not new.)^1^ For every 1 negative communication, 5 positive are required. Participants believed that communications should be balanced in covering health risks and the benefits that exist because the technologies supported by LDR exist. Personal opinions of experts surrounding the benefits and risks were identified as making an important contribution to peoples’ decision-making.

It is an important observation that most of the discussions that occurred in the focus groups reflected an understanding and alignment with what the literature has to say on risk perceptions. This suggests those in the space of science policy are at least aware of the challenges, if not taking action to overcome them. For example, although participants recognized that more work with the public is needed in relation to LDR and the LNT, it was thought more important to recognize that a value gap exists. Further, the context and form in which information is presented is just as important, if not more important, than the information itself.^23,24^ Recommendations included increasing communication of the hormesis theory and its evidence. More positive communication by nuclear experts and workers surrounding LDR, especially countering negative movies and public media is required. Trusted communicators are scientists and regulators, and Indigenous communities have specific contextual and cultural communication protocols. Although social media is generally regarded as untrustworthy, recommendations using the medium were thought especially important for accessing younger people. In order to do so, methods of communicating information appropriate for this demographic including interactive games and stories were proposed.

While focus groups never arrived at a specific solution for resolution of the LNT assumption that currently informs policy, several recommendations were made. These included augmenting communication of the hormesis theory and its evidence and engaging the international and national scientific community in a transparent and publicly engaged process for resolution of the LNT issue.

Although the literature rejects the deficit model (or the idea that people merely need to hear and internalize more scientific information), participants firmly believed that basic and ongoing education, especially starting with young people, was a necessity. Outlining societal benefits in respect of LDR and all its applications and manifestations (including in bananas) was considered essential. People make decisions every day to engage with, accept risks, and live in proximity of LDR. Understanding this just may be the key to advancing LDR science, medical diagnostics, and treatment.

## Notes

1 The Joint Statement of the IAEA et al confirms this reduction in diagnostic procedures and treatments. See https://www.iaea.org/sites/default/files/position_statement_final_endorsed.pdf

2 The Canadian Nuclear Safety and Control Act, S.C. 1987, C.9 is set by national radiation protection authorities and follows the United States’ National Council on Radiation Protection and Measurement which were issued in 1960 based on the precautionary principle and an invalid scientific LNT model
